# High-dose capsaicin exposure is associated with more severe inflammation, intestinal dysregulation, and IL-17 elevation in experimental autoimmune uveitis

**DOI:** 10.3389/fmicb.2026.1952362

**Published:** 2026-10-07

**Authors:** Tao Liu, Pei Liu, Mingzhu Liu, Jiawei Geng, Xiaoli Liu

**Affiliations:** Department of Ophthalmology, The Second Hospital of Jilin University, Changchun, Jilin, China

**Keywords:** capsaicin, experimental autoimmune uveitis, gut microbiota, kynurenic acid, tryptophan metabolism

## Abstract

**Purpose:**

To investigate whether capsaicin exposure increases the severity of experimental autoimmune uveitis (EAU) and to further characterize the associated alterations in intestinal homeostasis and immune regulation.

**Methods:**

Mice were orally administered different doses of capsaicin (CAP) from the day of EAU induction through the peak of disease on day 21. EAU severity was evaluated by fundus imaging, clinical scoring, and retinal histopathological scoring. Intestinal morphological changes were assessed by hematoxylin and eosin staining, while intestinal barrier integrity was evaluated by detecting colonic ZO-1 and MUC2 expression using immunofluorescence staining and enzyme-linked immunosorbent assays. IL-17A levels in the colon and spleen were quantified by ELISA. Fecal 16S rRNA gene sequencing, untargeted metabolomics, and colonic RNA sequencing were performed to characterize alterations in gut microbiota, metabolic profiles, and inflammatory pathways. Based on the identification of reduced kynurenic acid (KYNA) levels, KYNA co-treatment was subsequently performed to evaluate its effects on ocular inflammation, IL-17A levels, and intestinal barrier-related alterations during high-dose CAP exposure.

**Results:**

In the 80 mg/kg CAP group, retinal inflammation and tissue injury were more pronounced than in the other groups, accompanied by higher clinical and histopathological scores and increased IL-17A levels in the colon and spleen. High-dose CAP was also associated with ileal architectural abnormalities, reduced colonic mucosal thickness, and decreased colonic ZO-1 and MUC2 expression. 16S rRNA gene sequencing revealed alterations in gut microbial diversity and community structure. CAP-associated differential metabolites were enriched in the tryptophan metabolism pathway, and fecal KYNA levels were significantly reduced. KEGG over-representation analysis identified significant enrichment of the IL-17 signaling pathway among CAP-upregulated genes, whereas GSEA was not significant after multiple-testing correction, upregulation of *Il17a*, *Il17f*, and *Rorc*, and altered expression of genes involved in tryptophan metabolism. KYNA co-treatment reduced clinical and histopathologic scores, decreased IL-17A levels in the colon and spleen, and partially restored colonic ZO-1 and MUC2 expression.

**Conclusion:**

High-dose CAP exposure during EAU induction was associated with increased disease severity, accompanied by disrupted intestinal homeostasis, reduced fecal KYNA levels, and enhanced IL-17-related inflammation. KYNA co-treatment partially attenuated CAP-associated ocular and intestinal alterations.

## 1 Introduction

Uveitis is a sight-threatening intraocular inflammatory disease and represents one of the major causes of visual impairment worldwide (Tsirouki et al., 2018; Cunningham and Zierhut, 2021). The disease is characterized by a chronic and recurrent inflammatory course, and repeated episodes of intraocular inflammation can lead to irreversible damage to the retina, choroid, and other ocular tissues, ultimately resulting in visual dysfunction. Currently, dysregulated immune responses are considered a central factor in the pathogenesis and progression of uveitis, in which the imbalance between effector T-cell responses and regulatory T-cell-mediated immune control plays an important role. In particular, enhanced Th17-mediated inflammatory responses and impaired immune tolerance are recognized as key immunological features of autoimmune uveitis (Chi et al., 2007; Chi et al., 2008; Tang et al., 2009; Sun et al., 2015; Zhong et al., 2021; Zong et al., 2025).

Beyond local ocular immune abnormalities, accumulating evidence indicates that systemic immune regulation and intestinal homeostasis also contribute to the development and progression of uveitis. As a critical regulator of host immunity, the gut microbiota participates in disease regulation by influencing immune cell differentiation, inflammatory cytokine production, and autoreactive T-cell activation. In R161H mice, enhanced intestinal Th17 immune responses were associated with more severe experimental autoimmune uveitis (EAU), whereas depletion of the gut microbiota reduced both intestinal Th17 responses and ocular inflammation (Horai et al., 2015). Furthermore, certain commensal microorganisms may activate interphotoreceptor retinoid-binding protein (IRBP)-reactive autoreactive T cells through antigenic mimicry, thereby promoting autoimmune ocular inflammation (Zárate-Bladés et al., 2017; Wang et al., 2023). Clinical studies have also demonstrated altered gut microbial compositions in patients with active uveitis (Liu et al., 2024; Li et al., 2025). Moreover, fecal microbiota transplantation from patients with Vogt–Koyanagi–Harada (VKH) disease exacerbated EAU inflammation in recipient mice (Ye et al., 2020). Collectively, these findings suggest that gut microbial dysbiosis and microbiota-derived metabolites may contribute to the initiation and progression of uveitis by modulating systemic immune homeostasis (Nakamura et al., 2016; Salvador et al., 2023; Agrawal et al., 2025).

In recent years, dietary factors have attracted increasing attention as important environmental regulators of intestinal homeostasis and immune function. Capsaicin (CAP), the major pungent bioactive component of plants in the genus *Capsicum*, is a naturally occurring compound widely consumed in the human diet (Cordell and Araujo, 1993; Chen et al., 2019; Naves et al., 2019). Previous studies have demonstrated that CAP exhibits dose-related biological effects, with high-dose CAP inducing inflammatory injury in the jejunum, ileum, and colon, accompanied by alterations in neuropeptide signaling and microbiota-associated metabolic profiles (Xiang et al., 2022). In addition, excessive CAP exposure may impair intestinal barrier integrity, alter gut microbial composition, affect bile acid metabolism, and promote microbial component translocation, thereby establishing a pro-inflammatory intestinal microenvironment (Cheng et al., 2023). These findings suggest that dietary CAP exposure may regulate immune-mediated diseases through modulation of the gut–immune axis.

From a clinical perspective, during routine uveitis care, we observed that some patients reported recurrence or worsening of ocular inflammation after increasing their intake of chili peppers or other spicy foods. Chili consumption was also frequently reported among patients with uveitis in our clinical population. This clinical observation suggests that dietary factors may serve as potential environmental triggers influencing uveitis activity. However, whether CAP directly promotes EAU progression and whether its effects are associated with alterations in intestinal microbiota, metabolic homeostasis, and immune regulation remain unclear. Therefore, this study aimed to determine whether high-dose CAP is associated with greater disease severity in experimental autoimmune uveitis and to further investigate the underlying mechanisms involved.

## 2 Materials and methods

### 2.1 Induction of the experimental autoimmune uveitis

Female C57BL/6 J mice aged 6–8 weeks and weighing 22–26 g were purchased from Liaoning Changsheng Biotechnology Co., Ltd. (Liaoning, China). The mice were housed under specific pathogen-free (SPF) conditions and acclimatized for 1 week before the experiments. For the different-dose CAP experiment, mice were randomly assigned to the Control, EAU, EAU + 20 mg/kg CAP, EAU + 40 mg/kg CAP, and EAU + 80 mg/kg CAP groups. For the KYNA intervention experiment, the mice were randomly assigned to the EAU, EAU + KYNA, EAU + 80 mg/kg CAP, and EAU + 80 mg/kg CAP+KYNA groups, with 5–6 mice per group. EAU was induced as previously described (Caspi et al., 2008). Each mouse was subcutaneously immunized with an antigen emulsion containing 1,000 μg of interphotoreceptor retinoid-binding protein peptide 1–20 (IRBP₁–₂₀; amino acid sequence: GPTHLFQPSLVLDMAKVLLD; GL Biochem, Shanghai, China). The IRBP₁–₂₀ solution was thoroughly emulsified with an equal volume of complete Freund’s adjuvant (CFA; Cat. No. F5881; Sigma-Aldrich, St. Louis, MO, USA), resulting in a final concentration of 2.5 mg/mL heat-killed *Mycobacterium tuberculosis* in the emulsion. Pertussis toxin (PTX; Cat. No. 181; List Biological Laboratories, Campbell, CA, USA) was administered intraperitoneally at a dose of 0.5 μg per mouse on days 0 and 2 after immunization. On day 21 after immunization, fundus examinations were performed. Before examination, the mice were anesthetized by intraperitoneal injection of 1.25% 2,2,2-tribromoethanol (CAS No. 75–80-9; Shanghai Macklin Biochemical Co., Ltd., Shanghai, China) at a dose of 250 mg/kg. During the examination, the mice were placed on a heating pad to maintain body temperature and were continuously monitored until full recovery. Fundoscopic clinical scoring was performed as previously described to assess EAU severity (Agarwal et al., 2012). At the experimental endpoint, the mice were deeply anesthetized with 2,2,2-tribromoethanol. After a deep plane of anesthesia had been confirmed, the mice were euthanized by cervical dislocation before tissue collection. Death was confirmed by the absence of respiration and heartbeat. The eyes were subsequently collected for retinal histopathological examination, and histopathological scoring was performed as previously described to assess EAU severity (Agarwal et al., 2012). All animal experiments were approved by the Laboratory Animal Ethics Committee of Jilin University (approval No. SY202508037) and were conducted in accordance with the ARVO Statement for the Use of Animals in Ophthalmic and Vision Research and the ARRIVE guidelines.

### 2.2 Administration of capsaicin

Capsaicin (CAP; Cat. No. abs817025) was purchased from Absin Bioscience Inc. (Shanghai, China). CAP was first dissolved in dimethyl sulfoxide (DMSO; Cat. No. HY-Y0320; MedChemExpress, Monmouth Junction, NJ, USA) to prepare a stock solution and subsequently diluted to the required concentrations with corn oil (Cat. No. C805618; Macklin, Shanghai, China). From the day of EAU immunization (day 0) to day 21, mice in the EAU + 20 mg/kg CAP, EAU + 40 mg/kg CAP, and EAU + 80 mg/kg CAP groups received CAP once daily by oral gavage at doses of 20, 40, and 80 mg/kg, respectively, with a gavage volume of 10 mL/kg. Mice in the Control and EAU groups received an equivalent volume of the corresponding CAP vehicle once daily.

### 2.3 Administration of kynurenic acid

Kynurenic acid (KYNA; Cat. No. K3375; Sigma-Aldrich, St. Louis, MO, USA) was first dissolved in a small volume of DMSO to prepare a stock solution and subsequently diluted with sterile water to a final concentration of 1 mg/mL. From day 0 to day 21 after immunization, KYNA was administered once daily by oral gavage at a dose of 10 mg/kg with a gavage volume of 10 mL/kg. For the KYNA intervention experiment, mice were divided into four groups: EAU, EAU + KYNA, EAU + 80 mg/kg CAP, and EAU + 80 mg/kg CAP+KYNA groups. Mice in the EAU + KYNA group received KYNA together with the CAP vehicle; mice in the EAU + 80 mg/kg CAP group received CAP together with the KYNA vehicle; mice in the EAU + 80 mg/kg CAP+KYNA group received both CAP (80 mg/kg) and KYNA (10 mg/kg); and mice in the EAU group received corresponding volumes of both CAP and KYNA vehicles. All KYNA-related control groups received vehicle containing the same concentration and volume of DMSO as used for KYNA preparation.

### 2.4 Fecal DNA extraction and 16S rRNA gene sequencing

Fresh fecal samples were collected on day 21 from mice in the Control, EAU, and EAU + 80 mg/kg CAP groups (*n* = 5–6 per group) and stored at −80 °C. Total microbial DNA was extracted using the E. Z. N. A.® Soil DNA Kit (Omega Bio-Tek, USA; Cat. No. M5635-02). The V3–V4 region of the bacterial 16S rRNA gene was amplified using the 338F/806R primer pair. Following purification and library preparation, the amplicons were sequenced on an Illumina NovaSeq 6,000 platform by Personal Biotechnology Co., Ltd. (Shanghai, China). Sequence quality control, denoising, and chimera removal were performed using QIIME 2 (v2019.4) to generate amplicon sequence variants (ASVs). Taxonomic annotation, α-diversity, β-diversity, and microbial community composition analyses were subsequently performed.

### 2.5 Fecal metabolomic analysis

Fecal samples were collected on day 21 from mice in the Control, EAU, and EAU + 80 mg/kg CAP groups (*n* = 5–6 per group) and stored at −80 °C. Untargeted liquid chromatography–mass spectrometry (LC–MS)-based metabolomic analysis was performed by Personal Biotechnology Co., Ltd. (Shanghai, China). After metabolite extraction, chromatographic separation was carried out using an ACQUITY UPLC HSS T3 column, and mass spectrometric data were acquired in both positive- and negative-ion modes using an Orbitrap Exploris 120 mass spectrometer. Pooled quality-control samples were included to assess analytical stability. Raw data were processed using Compound Discoverer 3.3 for peak extraction, alignment, and normalization. Principal component analysis, differential metabolite analysis, and hierarchical clustering were performed in R. Metabolic pathway annotation and enrichment analysis were conducted using the Kyoto Encyclopedia of Genes and Genomes (KEGG) database.

### 2.6 Intestinal morphology

On day 21, the mice were euthanized, and ileal and colonic tissues were collected and immediately rinsed thoroughly with ice-cold phosphate-buffered saline (PBS) to remove luminal contents. The tissues were then fixed in 4% paraformaldehyde tissue fixative (Servicebio, Wuhan, China) at 4 °C, routinely dehydrated, cleared, and embedded in paraffin. Paraffin-embedded sections were deparaffinized in xylene, rehydrated through a graded ethanol series, and stained with hematoxylin and eosin (H&E). Microscopic images were acquired using a Leica DM5000B microscope (Leica Microsystems, Wetzlar, Germany). For each mouse, three randomly selected ileal fields from different tissue sections were analyzed. Villus length, crypt depth, submucosal thickness, and muscularis thickness were measured using ImageJ software (National Institutes of Health, Bethesda, MD, USA). Colonic mucosal, submucosal, and muscularis thicknesses were measured using the same procedure.

### 2.7 RNA-seq analysis of intestinal tissue

Colonic tissues from mice in the EAU and EAU + 80 mg/kg CAP groups (*n* = 3 per group) were subjected to RNA sequencing (RNA-seq). Total RNA extraction, mRNA enrichment, library construction, and sequencing were performed by Novogene Co., Ltd. (Beijing, China). Libraries were sequenced on an Illumina platform using a 150-bp paired-end (PE150) strategy. After quality control, clean reads were aligned to the mouse reference genome using HISAT2, and gene expression was quantified using feature Counts. Differential expression analysis was performed using DESeq2. Differentially expressed genes were identified using thresholds of Benjamini–Hochberg-adjusted *p* ≤ 0.05 and |log₂ fold change| ≥ 1. Kyoto Encyclopedia of Genes and Genomes (KEGG) pathway enrichment analysis was performed for genes upregulated in the CAP group, with enrichment *p* values adjusted using the Benjamini–Hochberg method. Gene set enrichment analysis (GSEA) was conducted using all genes ranked in descending order according to log₂ fold change, with mouse KEGG pathway gene sets as the reference, to evaluate the overall enrichment pattern of the IL-17 signaling pathway.

### 2.8 Enzyme-linked immunosorbent assay

Colonic and splenic tissues were collected for IL-17A measurement, whereas colonic tissues were used for ZO-1 and MUC2 detection. Tissues from mice in the Control, EAU, EAU + 80 mg/kg CAP, and EAU + 80 mg/kg CAP+KYNA groups (*n* = 6 per group) were analyzed using ELISA according to the manufacturers’ instructions. Briefly, tissues were homogenized in ice-cold phosphate-buffered saline (PBS) at a ratio of 100 mg tissue per 1 mL PBS and centrifuged, and the supernatants were collected for subsequent analysis.

IL-17A levels were measured using a mouse IL-17A ELISA kit (Proteintech, Wuhan, China). ZO-1 and MUC2 levels were determined using the corresponding ELISA kits (Jiangsu Meimian Industrial Co., Ltd., China). Protein concentrations were calculated based on the respective standard curves.

### 2.9 Immunofluorescence staining

Colonic tissues from mice in the Control, EAU, and EAU + 80 mg/kg CAP groups (*n* = 3 per group) were subjected to immunofluorescence staining. Paraffin-embedded colonic sections were deparaffinized and rehydrated through a graded ethanol series. Heat-induced antigen retrieval was performed in EDTA antigen retrieval buffer at 100 °C for 15 min. After cooling to room temperature, the sections were washed with phosphate-buffered saline (PBS). Endogenous peroxidase activity was blocked by incubation with 3% hydrogen peroxide at room temperature in the dark, followed by blocking with bovine serum albumin (BSA) to reduce nonspecific binding. The sections were first incubated overnight at 4 °C in a humidified chamber with a primary antibody against ZO-1 (GB151981; Servicebio, China; 1:5,000). After thorough washing with PBS, the sections were incubated with a horseradish peroxidase-conjugated secondary antibody, followed by fluorescent labeling using a tyramide signal amplification (TSA) system. The first-round antibody complexes were then removed using an antibody stripping solution. A second round of immunofluorescence staining was performed using an anti-MUC2 antibody (GB15344; Servicebio, China; 1:10,000), followed by TSA labeling using the same procedure, thereby enabling dual immunofluorescence detection of ZO-1 and MUC2. Nuclei were counterstained with DAPI, and the sections were mounted using an antifade mounting medium. Fluorescence images of colonic tissues from the different groups were acquired using a fluorescence microscope, and fluorescence signals were quantified using Saiviewer software. For each mouse, three nonconsecutive colonic sections were selected, and three randomly chosen, nonoverlapping fields were analyzed per section. Quantification was performed using identical microscope exposure settings and image-analysis thresholds. The mean value of the nine fields from each mouse was calculated and treated as one independent biological replicate. ZO-1 and MUC2 expression in the colonic mucosa was evaluated as the percentage of positively stained area.

### 2.10 Statistical analysis

Statistical analyses were performed using SPSS version 24.0. Continuous variables were first assessed for normality and homogeneity of variance. Normally distributed data are presented as the mean ± standard deviation or mean ± standard error of the mean. Comparisons between two groups were performed using an unpaired Student’s *t*-test when variances were equal or Welch’s *t*-test when variances were unequal. For comparisons among three or more groups, one-way analysis of variance followed by Tukey’s *post hoc* multiple-comparisons test was used when the assumption of homogeneity of variance was met; otherwise, Welch’s analysis of variance followed by the Games–Howell *post hoc* test was applied. Non-normally distributed data were analyzed using the Mann–Whitney *U* test or Kruskal–Wallis test, followed by Dunn’s multiple-comparisons test when appropriate. Correlations between microbial taxa and metabolites were evaluated using Spearman’s rank correlation analysis, with multiple-testing correction performed using the Benjamini–Hochberg method. All tests were two-sided, and *p* < 0.05 was considered statistically significant. Figures were generated using GraphPad Prism version 10.0 (GraphPad Software, San Diego, CA, USA).

## 3 Results

### 3.1 High-dose CAP is associated with increased EAU severity

To evaluate the effects of CAP on EAU severity, fundus changes, retinal histopathology, and IL-17A levels in the spleen and colon were assessed after treatment with different doses of CAP. Fundus examination showed no obvious abnormalities in the Control group, whereas EAU mice exhibited retinal vascular dilation, tortuosity, and scattered inflammatory lesions, which were more pronounced changes after 80 mg/kg CAP administration (Figure 1A). H&E staining revealed retinal structural disruption and focal folding in EAU mice, with more pronounced damage observed in the 80 mg/kg CAP group (Figure 1B). Compared with vehicle-treated EAU mice, only mice treated with 80 mg/kg CAP exhibited significantly higher clinical and histopathological scores, whereas low-dose CAP treatment (20 and 40 mg/kg) had limited effects on disease severity (Figures 1C,D). Moreover, 80 mg/kg CAP further increased IL-17A levels in both the colon and spleen (Figures 1E,F).

**Figure 1:**
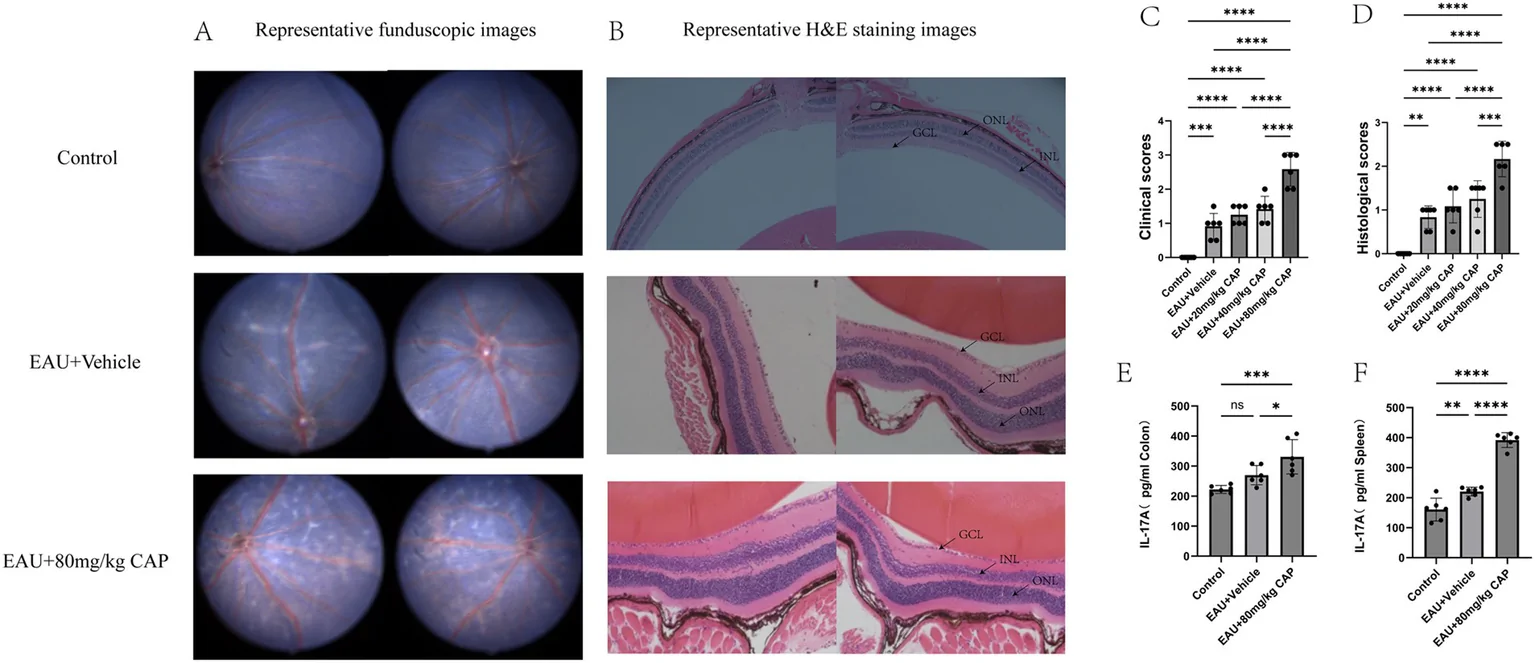
High-dose capsaicin is associated with increased ocular inflammation and IL-17A levels in EAU mice. **(A)** Representative fundus images of mice in each group on day 21 after EAU induction. **(B)** Representative hematoxylin and eosin-stained retinal sections. **(C,D)** Clinical and histopathological scores in the Control, EAU + Vehicle, and different-dose CAP groups. **(E,F)** IL-17A levels in colon and spleen tissues from the Control, EAU + Vehicle, and EAU + 80 mg/kg CAP groups, as determined by ELISA. Data are presented as the mean ± SEM. Each data point represents one mouse; *n* = 5–6 per group. Statistical comparisons were performed using one-way analysis of variance followed by Tukey’s multiple-comparisons test. ns, not significant; *p < 0.05, **p < 0.01, ***p < 0.001, and ****p < 0.0001. CAP, capsaicin; EAU, experimental autoimmune uveitis; IL-17A, interleukin-17A.

Together with the ocular findings, these results indicated that 20 and 40 mg/kg CAP had limited effects on the overall EAU phenotype, whereas 80 mg/kg CAP was associated with more pronounced ocular inflammation and intestinal alterations. Therefore, subsequent microbiota, metabolomic, transcriptomic, and KYNA co-treatment experiments focused on the 80 mg/kg CAP group.

### 3.2 High-dose CAP treatment is associated with gut microbiota alterations in EAU mice

To investigate gut microbial alterations associated with high-dose CAP exposure in EAU, 16S rRNA gene sequencing was performed to characterize microbial diversity and composition after CAP treatment. Alpha-diversity analysis showed no significant difference in the Chao1 index among the three groups (*p* = 0.36), whereas Simpson and Shannon indices differed significantly (*p* = 0.015 and *p* = 0.023, respectively) (Figure 2A). Principal coordinate analysis (PCoA) revealed a separation trend among the Control, EAU, and CAP groups, with PC1 and PC2 explaining 36.5 and 14.7% of the total variation, respectively (Figure 2B). At the phylum level, *Bacteroidota* and Firmicutes_D were the predominant taxa across all groups. Compared with the Control group, EAU mice showed increased *Bacteroidota* and decreased Firmicutes_D abundance, whereas CAP treatment further altered microbial composition with an increase in *Actinobacteriota* abundance (Figure 2C). At the genus level, several taxa, including *Ligilactobacillus*, *Muribaculum*, CAG-485, *Allobaculum*, *Duncaniella*, *Paramuribaculum,* and *Bifidobacterium*, varied in relative abundance among groups (Figure 2D). LEfSe analysis identified distinct microbial signatures, with *Ligilactobacillus-*enriched taxa in the Control group, *Erysipelotrichaceae*-related taxa in the EAU group, and *Bifidobacterium*, *Bifidobacteriaceae*, *Actinobacteriota*, and *Dubosiella* enriched in the CAP group (Figure 2E).

**Figure 2:**
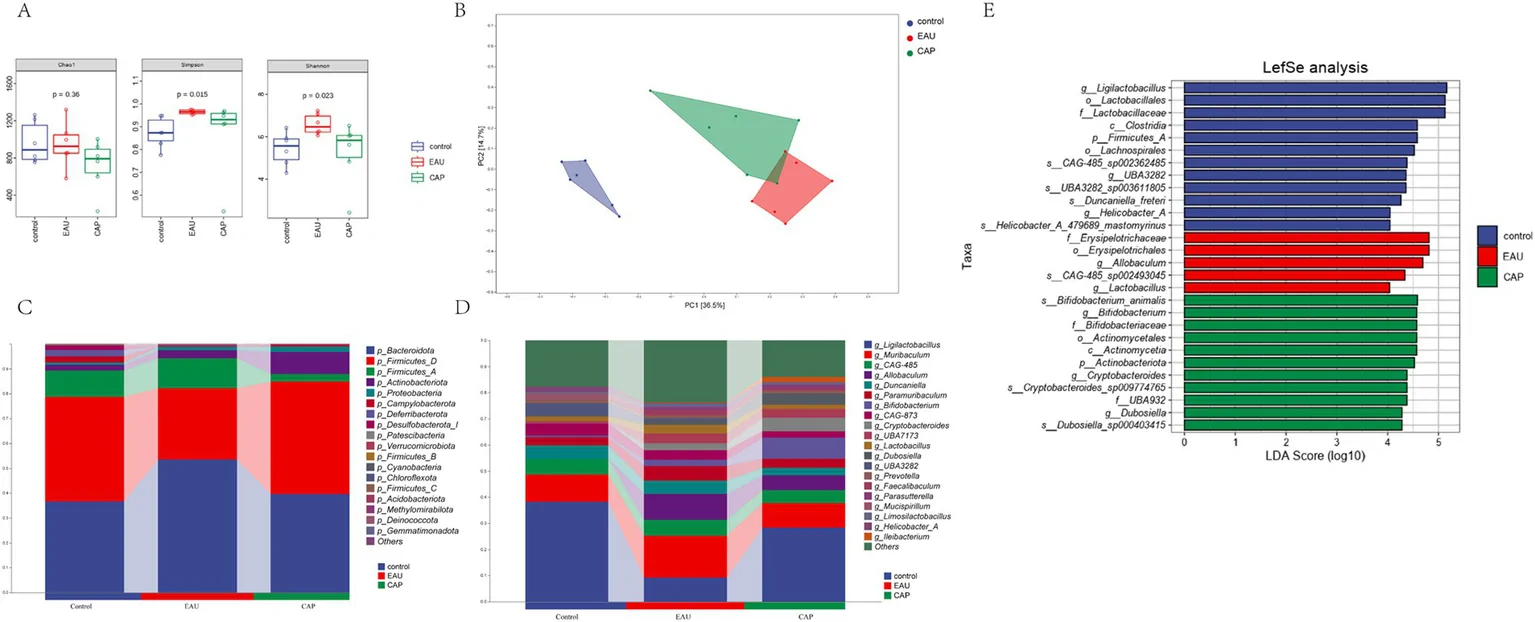
High-dose capsaicin exposure is associated with altered gut microbial community structure in EAU mice. **(A)** α-Diversity of the gut microbiota assessed using the Chao1, Simpson, and Shannon indices. *p* values are indicated in the plots. **(B)** Principal coordinate analysis based on Bray–Curtis distances showing differences in microbial community composition among the Control, EAU, and CAP groups. PC1 and PC2 explained 36.5 and 14.7% of the total variation, respectively. **(C,D)** Relative abundance and community composition of the predominant bacterial taxa at the phylum and genus levels, respectively. **(E)** Linear discriminant analysis effect size analysis showing the bacterial taxa enriched in each group. Taxa with an LDA score >4.0 are shown. CAP, EAU mice treated with 80 mg/kg capsaicin; EAU, experimental autoimmune uveitis; PCoA, principal coordinate analysis; LEfSe, linear discriminant analysis effect size; LDA, linear discriminant analysis.

### 3.3 High-dose CAP exposure is associated with reduced fecal kynurenic acid levels and altered tryptophan metabolism in EAU mice

To further investigate fecal metabolic alterations accompanying high-dose CAP exposure in EAU, untargeted metabolomic analysis was performed. The metabolomic profiles showed high within-group correlations and relatively lower correlations between groups (Figure 3A). PCA demonstrated a separation trend among the Control, EAU, and CAP groups (Figure 3B). KEGG pathway enrichment analysis revealed that differential metabolites among the three groups were mainly enriched in the tryptophan metabolism pathway (Figure 3C). Further analysis showed a decreasing trend in kynurenic acid (KYNA) levels after CAP treatment. Quantitative analysis demonstrated that KYNA levels were significantly reduced in the CAP group compared with both the Control and EAU groups (Figures 3D,E). Moreover, differential microbial taxa were significantly correlated with tryptophan-related differential metabolites, suggesting that CAP- associated gut microbial remodeling may be related to altered tryptophan metabolism (Figure 3F).

**Figure 3:**
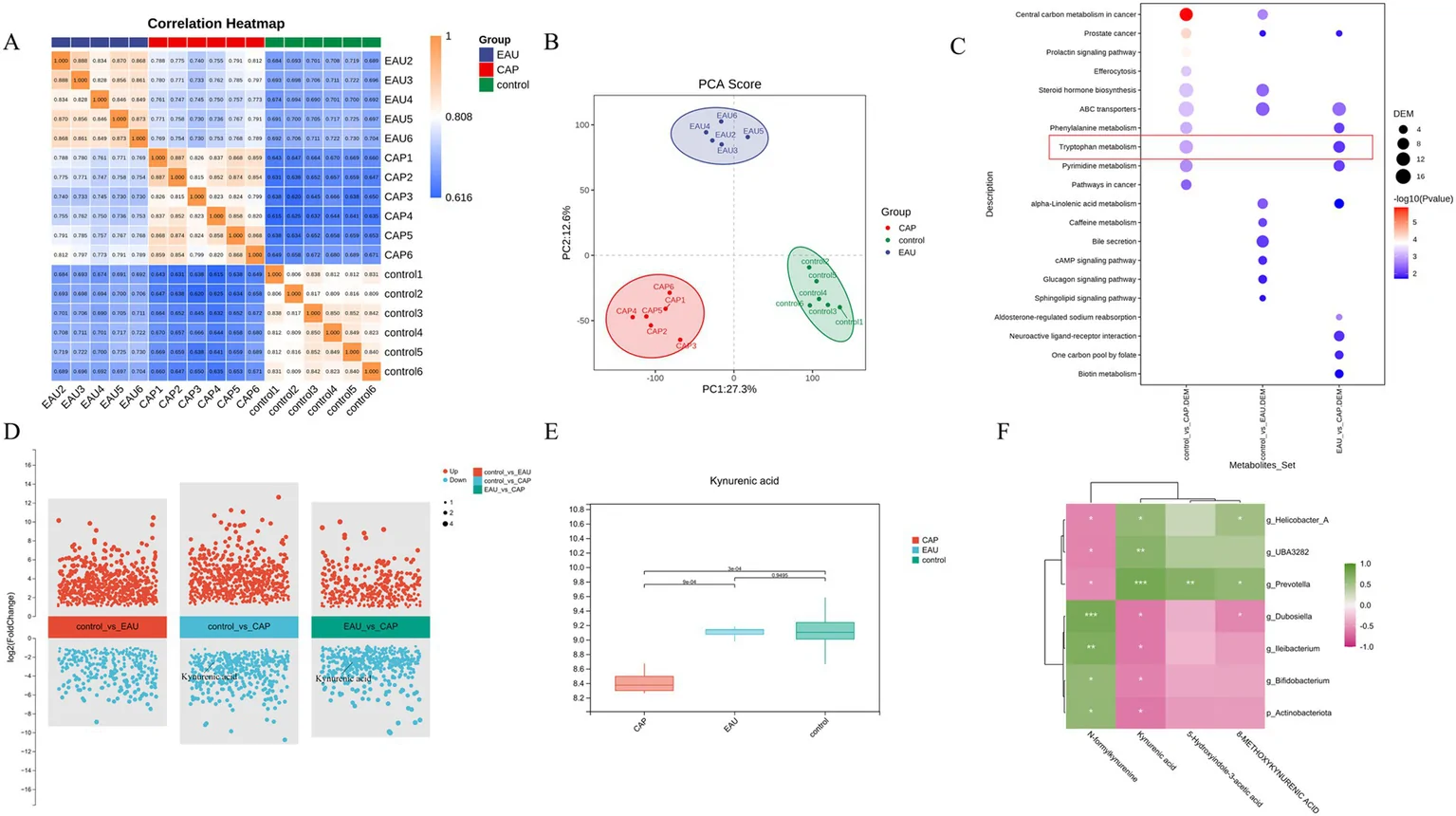
High-dose capsaicin exposure is associated with altered fecal metabolic profiles and reduced kynurenic acid levels in EAU mice. **(A)** Sample-to-sample correlation heatmap based on fecal metabolite abundance profiles. **(B)** Principal component analysis of the fecal metabolome in the Control, EAU, and CAP groups. PC1 and PC2 explained 27.3 and 12.6% of the total variance, respectively. **(C)** KEGG pathway enrichment analysis of differential metabolites identified in the Control versus EAU, Control versus CAP, and EAU versus CAP comparisons. Bubble size represents the number of differential metabolites, and color indicates −log10(*p* value). **(D)** Distribution of upregulated and downregulated metabolites in each pairwise comparison; kynurenic acid is highlighted. **(E)** Relative abundance of kynurenic acid in the three groups. *p* values are indicated in the plot. **(F)** Spearman correlation heatmap between selected differential microbial taxa and tryptophan-related metabolites. Green and magenta indicate positive and negative correlations, respectively. Asterisks indicate FDR-adjusted statistical significance: *p < 0.05, **p < 0.01, and ***p < 0.001. CAP, EAU mice treated with 80 mg/kg capsaicin; EAU, experimental autoimmune uveitis; PCA, principal component analysis; KEGG, Kyoto Encyclopedia of Genes and Genomes; DEM, differential metabolite; FDR, false discovery rate.

### 3.4 High-dose CAP exposure is associated with intestinal architecture and reduced the expression of intestinal mucosal barrier proteins in EAU mice

To evaluate the effects of different doses of CAP on intestinal morphology in EAU mice, ileal and colonic tissues were examined by H&E staining and morphometric analysis (Figures 4A–D). Intestinal morphological changes varied across CAP dose groups. In the ileum, compared with the EAU group, 20 mg/kg CAP increased villus height, whereas higher doses (40 and 80 mg/kg) reduced villus height. CAP administration also increased crypt depth, with 80 mg/kg CAP producing more pronounced structural alterations (Figures 4A,B). In the colon, CAP treatment was primarily associated with reduced mucosal thickness (Figures 4C,D).

**Figure 4:**
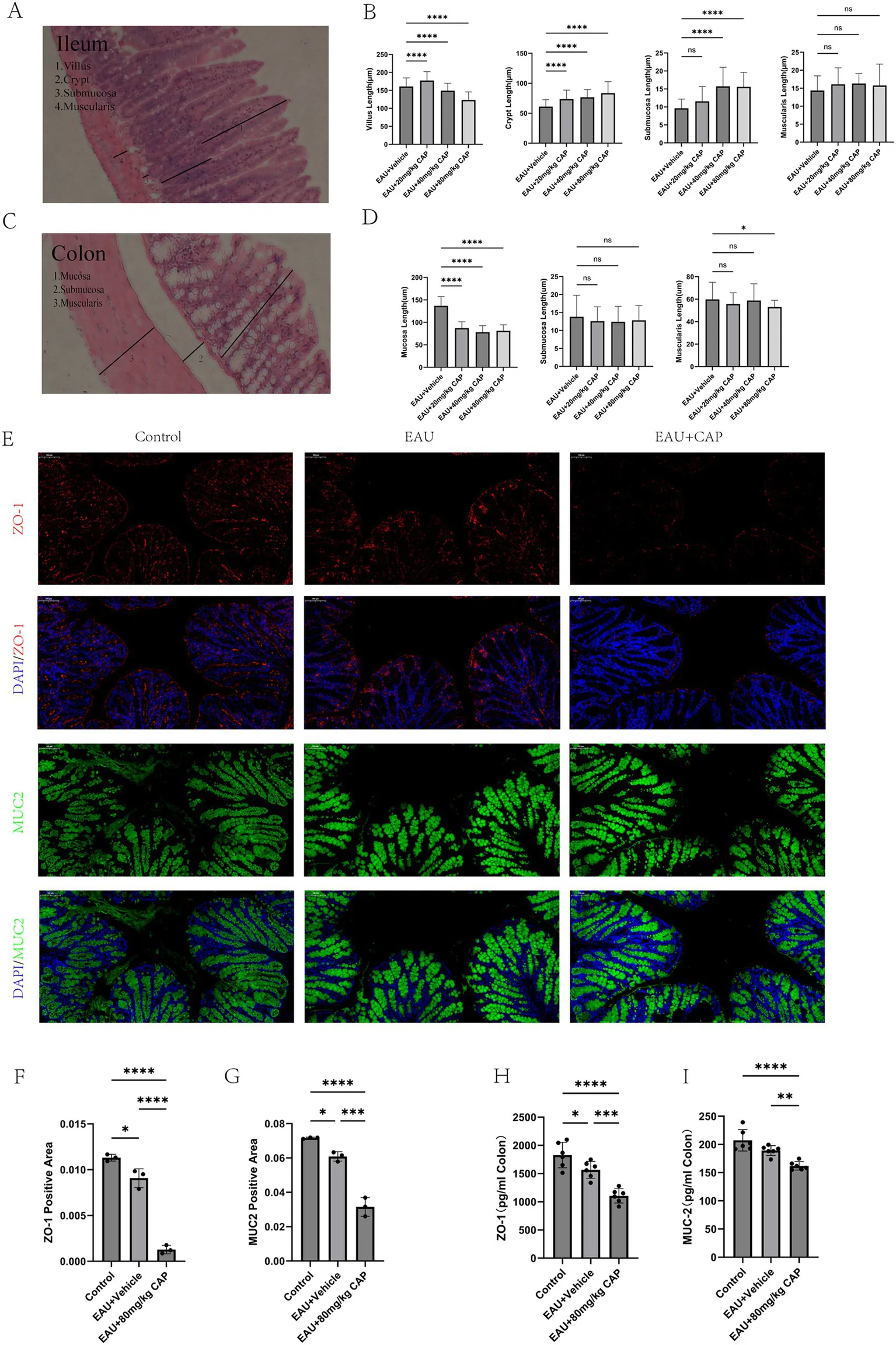
High-dose capsaicin exposure is associated with intestinal morphological alterations and reduced colonic barrier-associated markers in EAU mice. **(A)** Representative hematoxylin and eosin (H&E)-stained ileal section illustrating the measurement of villus length, crypt depth, submucosal thickness, and muscularis thickness. **(B)** Quantification of ileal villus length, crypt depth, submucosal thickness, and muscularis thickness in the EAU + Vehicle and different-dose CAP groups. **(C)** Representative H&E-stained colonic section illustrating the measurement of mucosal, submucosal, and muscularis thickness. **(D)** Quantification of colonic mucosal, submucosal, and muscularis thickness. **(E)** Representative immunofluorescence images of ZO-1 and MUC2 in colonic tissues from the Control, EAU + Vehicle, and EAU + 80 mg/kg CAP groups. ZO-1 is shown in red, MUC2 in green, and nuclei counterstained with DAPI in blue. **(F,G)** Quantification of the ZO-1- and MUC2-positive areas. **(H,I)** ZO-1 and MUC2 levels in colonic tissues measured by ELISA. Data are presented as the mean ± SEM. Each data point represents one mouse. Statistical comparisons were performed using one-way analysis of variance followed by Tukey’s multiple-comparisons test. ns, not significant; *p < 0.05, **p < 0.01, ***p < 0.001, and ****p < 0.0001. CAP, capsaicin; EAU, experimental autoimmune uveitis; ZO-1, zonula occludens-1; MUC2, mucin 2; DAPI, 4′,6-diamidino-2-phenylindole.

Immunofluorescence staining and ELISA were further performed to assess intestinal barrier integrity. In Control mice, ZO-1 exhibited continuous localization along the colonic mucosa, whereas ZO-1 expression was reduced in EAU mice and further decreased with discontinuous distribution after 80 mg/kg CAP treatment. Quantitative analysis showed that the ZO-1-positive area was significantly reduced in the EAU group and further decreased after CAP administration (Figures 4E,F). Similarly, MUC2 fluorescence intensity and positive area were reduced in EAU mice and further decreased in the EAU + 80 mg/kg CAP group (Figures 4E,G). ELISA confirmed reduced colonic ZO-1 levels in EAU mice, with a further decrease following CAP treatment, whereas MUC2 levels showed no significant difference between Control and EAU groups but were significantly reduced after CAP administration (Figures 4H,I).

### 3.5 High-dose CAP treatment is associated with colonic transcriptomic changes and IL-17-related signatures

To further characterize the transcriptional alterations associated with CAP exposure in EAU, RNA sequencing was performed on colonic tissues. PCA showed a separation trend between the CAP and EAU groups (Figure 5A). Differential expression analysis identified 585 differentially expressed genes (DEGs), including 265 upregulated and 320 downregulated genes (Figure 5B). KEGG enrichment analysis of CAP-upregulated genes identified significant over-representation of the IL-17 signaling pathway (GeneRatio = 5/42, gene count = 5, adjusted *p* = 0.012235), with S100a9, Lcn2, Ccl20, S100a8, and Cxcl5 contributing to this enrichment (Figure 5C). In contrast, GSEA of the full ranked gene list showed a positive enrichment pattern for the IL-17 signaling pathway (NES = 1.38, nominal *p* < 0.001), but this enrichment was not statistically significant after multiple-testing correction (FDR q = 0.984) (Figure 5D). The discrepancy between the ORA and GSEA results may indicate that a relatively small subset of strongly altered genes drives the apparent IL-17 signal, rather than coordinated pathway regulation. At the individual-gene level, compared with the EAU group, CAP treatment increased the expression of inflammatory genes Il17a, Il17f, and Rorc, as well as tryptophan metabolism-related genes Tdo2 and Tph1. In contrast, genes associated with intestinal immune homeostasis, including Ahrr, Il22, and Cyp1a1, were downregulated in the CAP group (Figure 5E).

**Figure 5:**
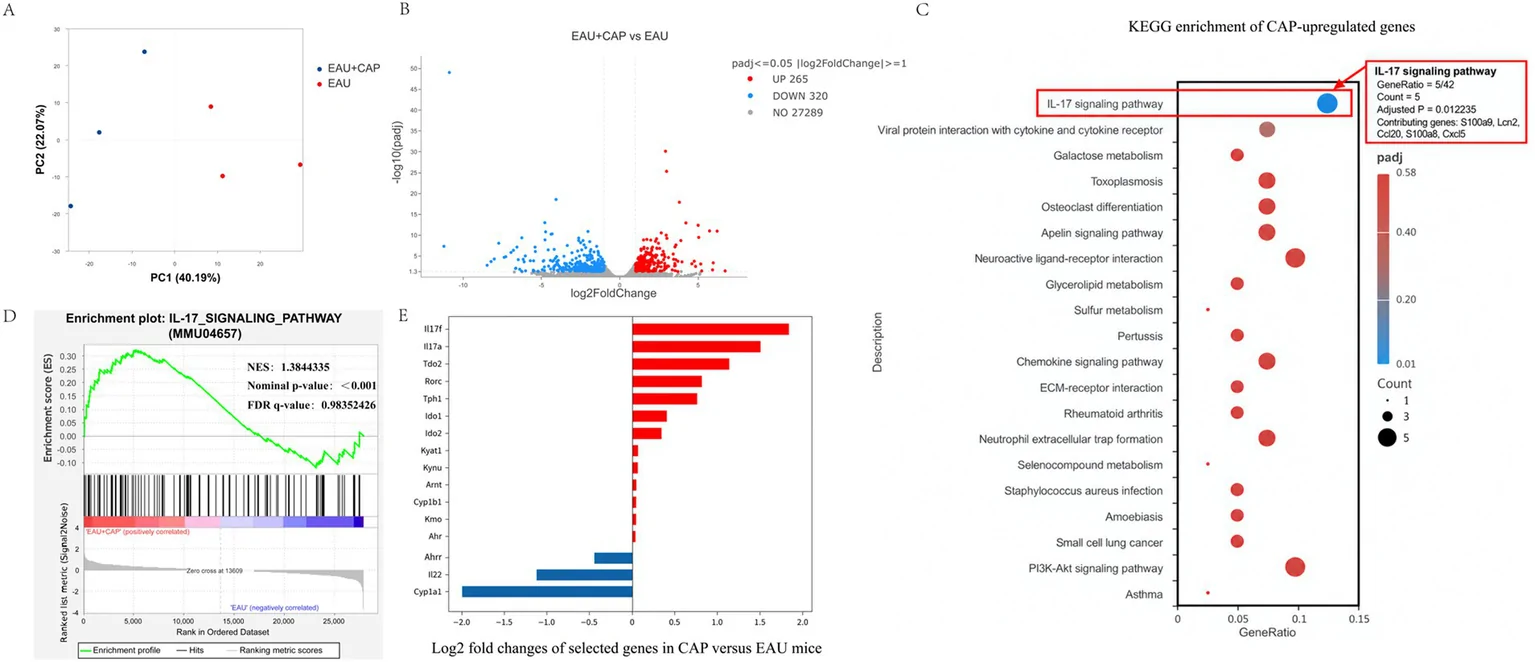
High-dose capsaicin exposure is associated with colonic gene-expression changes and IL-17-related inflammatory signatures in EAU mice. **(A)** Principal component analysis of colonic transcriptomes from the EAU and CAP groups (*n* = 3 per group). PC1 and PC2 explained 40.19 and 22.07% of the total variance, respectively. **(B)** Volcano plot showing differentially expressed genes between the CAP and EAU groups. Red and blue dots represent upregulated and downregulated genes, respectively, based on BH-adjusted *p* ≤ 0.05 and |log₂ fold change| ≥ 1. **(C)** KEGG pathway enrichment analysis of genes upregulated in the CAP group. Bubble size represents the number of enriched genes, and color indicates the adjusted *p* value. **(D)** GSEA of the IL-17 signaling pathway in the EAU + 80 mg/kg CAP versus EAU comparison. **(E)** Log₂ fold changes of selected genes associated with IL-17 signaling, tryptophan metabolism, and AhR-related mucosal responses. Red and blue bars indicate increased and decreased expression in the CAP group, respectively. CAP, EAU mice treated with 80 mg/kg capsaicin; EAU, experimental autoimmune uveitis; PCA, principal component analysis; KEGG, Kyoto Encyclopedia of Genes and Genomes; GSEA, gene set enrichment analysis; NES, normalized enrichment score; FDR, false discovery rate; AhR, aryl hydrocarbon receptor.

### 3.6 KYNA co-treatment is associated with reduced EAU severity and altered intestinal homeostasis during high-dose CAP exposure

To investigate the role of KYNA in CAP exposure in EAU, KYNA co-treatment was performed in CAP-treated mice. Fundus examination showed marked retinal vascular dilation, tortuosity, and multiple inflammatory lesions in the EAU + 80 mg/kg CAP group, whereas these abnormalities were attenuated in the KYNA co-treatment group compared with the EAU + 80 mg/kg CAP group (Figure 6A). Consistent with the fundus findings, retinal H&E staining revealed structural disruption, focal folding, and inflammatory cell infiltration after CAP administration, which were alleviated by KYNA co-treatment (Figure 6B). Compared with the other groups, the EAU + 80 mg/kg CAP group exhibited significantly higher clinical and histopathological scores. KYNA treatment significantly reduced both scores, although they remained higher than those in the EAU + KYNA group (Figures 6C,D). Furthermore, KYNA significantly reduced CAP-induced elevation of IL-17A levels in both the colon and spleen. Colonic IL-17A levels were restored to levels comparable with those in the EAU group, whereas splenic IL-17A levels remained higher than those in the EAU group (Figures 6E,F). In addition, KYNA co-treatment improved CAP-induced intestinal barrier impairment, as indicated by restored colonic ZO-1 and MUC2 expression (Figures 6G,H).

**Figure 6:**
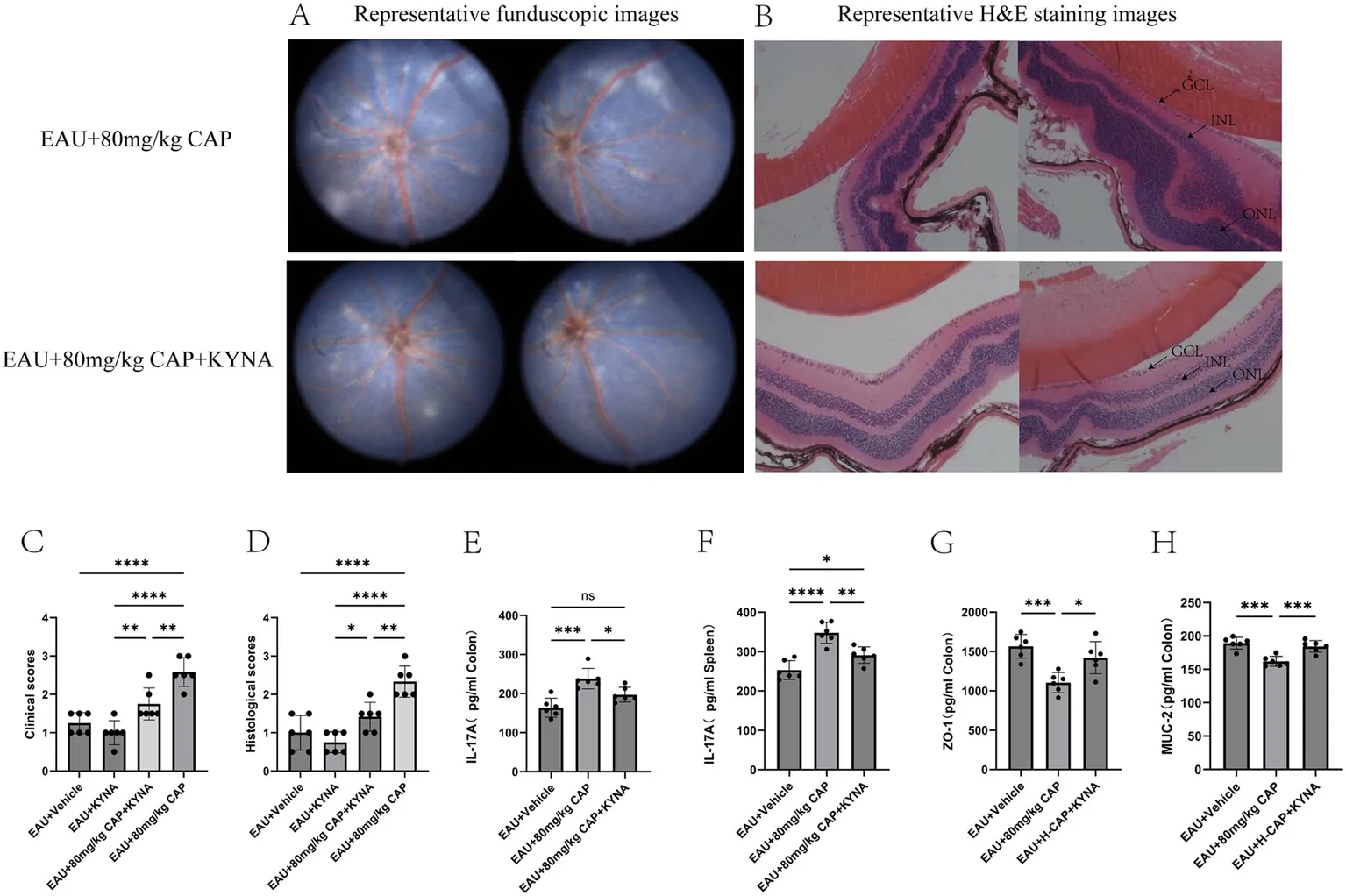
Kynurenic acid partially attenuates high-dose capsaicin-associated increases in EAU severity. **(A)** Representative fundoscopic images of mice in the EAU + 80 mg/kg CAP and EAU + 80 mg/kg CAP+KYNA groups on day 21 after EAU induction. **(B)** Representative hematoxylin and eosin-stained retinal sections from the indicated groups. **(C,D)** Clinical and histopathological scores in the EAU + Vehicle, EAU + KYNA, EAU + 80 mg/kg CAP+KYNA, and EAU + 80 mg/kg CAP groups. **(E,F)** IL-17A levels in colon and spleen tissues from the EAU + Vehicle, EAU + 80 mg/kg CAP, and EAU + 80 mg/kg CAP+KYNA groups, as determined by ELISA. **(G,H)** ZO-1 and MUC2 levels in colonic tissues measured by ELISA. Data are presented as the mean ± SEM. Each data point represents one mouse; *n* = 5–6 per group. Statistical comparisons were performed using one-way analysis of variance followed by Tukey’s multiple-comparisons test. ns, not significant; *p < 0.05, **p < 0.01, ***p < 0.001, and ****p < 0.0001. CAP, capsaicin; EAU, experimental autoimmune uveitis; KYNA, kynurenic acid; IL-17A, interleukin-17A; ZO-1, zonula occludens-1; MUC2, mucin 2.

## 4 Discussion

The present study showed that high-dose CAP (80 mg/kg) was associated with more pronounced ocular inflammation and retinal tissue damage in EAU mice, accompanied by increased IL-17A levels in the spleen and colon. Integrated analyses of histology, 16S rRNA sequencing, untargeted metabolomics, colonic transcriptomics, and KYNA intervention indicated that high-dose CAP was associated with intestinal barrier disruption, gut microbial and metabolic alterations, and enhanced IL-17-related inflammation. These findings suggest that high-dose CAP exposure is associated with greater disease severity，intestinal dysregulation and increased IL-17A levels in EAU.

Gut microbial dysbiosis is considered to contribute to the development and progression of uveitis (Nakamura et al., 2014; Janowitz et al., 2019). In the present study, 16S rRNA sequencing demonstrated that high-dose CAP markedly altered the overall gut microbial structure in EAU mice. LEfSe analysis identified several taxa enriched in the CAP group, including *Bifidobacterium*, *Actinobacteriota*, *Cryptobacteroides*, UBA932, and *Dubosiella*. Similar results were reported previously, A greater abundance of *Bifidobacterium* was also observed in high-dose CAP-treated mice (Xiang et al., 2022). Increased *Bifidobacterium* abundance has also been reported in patients with active ulcerative colitis (Wang et al., 2014) and Behcet’s disease (Shimizu et al., 2016). *Bifidobacterium* is commonly considered a beneficial probiotic genus. However, it was reported that *Bifidobacterium* could influence Th17-, Th1-, or Treg-associated immune responses (Lopez et al., 2011; Tan et al., 2016). Therefore, the role of *Bifidobacterium* still needs to be studied. It is worth noting that, given the absence of a non-EAU + high-dose CAP group, the intestinal abnormalities maybe existed as parallel changes associated with high-dose CAP exposure in EAU rather than as evidence that intestinal disruption mediates the increase in EAU severity.

High-dose CAP exposure in EAU mice was accompanied by alterations in the intestinal metabolic profile. PCA showed a separation trend between the EAU + 80 mg/kg CAP and EAU groups, indicating differences in fecal metabolite composition. KEGG pathway enrichment analysis revealed that CAP-associated differential metabolites were mainly enriched in the tryptophan metabolism pathway. Further analysis showed that KYNA levels were significantly reduced after high-dose CAP treatment compared with both the EAU and Control groups, suggesting that CAP affects the KYNA-related branch of the kynurenine pathway. Previous studies have shown that CAP alters tryptophan metabolism and reduces KYNA levels in different disease models (Li et al., 2022; Zhang et al., 2025). Tryptophan is metabolized through several pathways, including the kynurenine, serotonin, and microbial indole pathways, and its metabolites contribute to intestinal mucosal homeostasis and immune regulation (Seo and Kwon, 2023; Xue et al., 2023). As a key metabolite of the kynurenine pathway, KYNA may regulate inflammatory responses through receptors such as GPR35. Previous studies have reported that KYNA suppresses lipopolysaccharide-induced TNF-α release and inhibits NLRP3 inflammasome activation and IL-1β production through GPR35 signaling (Wang et al., 2006; Sun et al., 2022). Therefore, reduced KYNA levels accompanied high-dose CAP exposure, together with increased intestinal inflammatory activity and greater EAU severity.

The mechanism of high dose CAP exposure produced increased EAU severity was unclear. Our results showed that all three different doses of CAP significantly damaged the intestinal mucosa, but not all doses were associated with ocular inflammation. This suggested that physical disruption of the intestinal mucosa was not the only factor contributing to uveitis, Immune responses might also be involved. Higher IL-17 transcriptomic levels were observed in colon and spleen of high-dose CAP group. This suggested that high-dose CAP could induce more severe inflammation in the intestine and peripheral tissues. In previous studies, higher levels of IL-17 in the spleen were found to be related to active ocular inflammation (Amadi-Obi et al., 2007; Escobar et al., 2013). Therefore, we speculated that high-dose CAP was involved in the increased severity of EAU by inducing IL-17 expression. More experiments need to be performed in the future to investigate the cellular source of IL-17 and demonstrate whether IL-17 is required for CAP-induced worsening of EAU.

In EAU mice treated with 80 mg/kg CAP, KYNA co-treatment reduced fundus inflammation, decreased clinical and histopathological scores, lower IL-17A levels in the colon and spleen, and higher colonic ZO-1 and MUC2 expression. These findings indicate that exogenous KYNA partially attenuates the increased EAU severity associated with high-dose CAP exposure suggesting that reduced KYNA availability may represent a metabolically relevant alteration associated with in this process. Further analysis revealed increased expression trends of tryptophan metabolism-related genes, including Tdo2, Tph1, Ido1, and Ido2, in colonic transcriptomes, suggesting that CAP may influence both kynurenine and serotonin metabolic pathways. TDO2 catalyzes the conversion of tryptophan to kynurenine, which can activate AhR signaling. AhR contributes to intestinal immune homeostasis by regulating IL-22 production, tight junction expression, and epithelial barrier function (Lee et al., 2011; Opitz et al., 2011; Yu et al., 2018). KYNA, generated from kynurenine by kynurenine aminotransferases, is a key metabolite of the kynurenine pathway, and its intestinal levels are shaped by the combined effects of host metabolic enzymes and gut microbial metabolism (Rossi et al., 2004; Miyamoto et al., 2023). Previous studies have shown that KYNA can regulate inflammatory responses through AhR- and GPR35-associated pathways, suppress NLRP3 inflammasome activation, and promote intestinal epithelial barrier integrity (Wang et al., 2006; Wang et al., 2021; Sun et al., 2022; Wang et al., 2022).

These findings suggest that alterations in tryptophan metabolism and AhR/IL-22-related mucosal homeostasis may accompany intestinal inflammatory and barrier abnormalities during high-dose CAP exposure (Zelante et al., 2013; Schiering et al., 2017). Notably, KYNA co-treatment only partially, rather than completely, attenuated the CAP-associated increase in disease severity, suggesting that additional factors, including other tryptophan metabolites, non-tryptophan metabolic alterations, intestinal tissue injury, and immune-cell responses, may collectively contribute to this process. Tryptophan metabolism involves host kynurenine and serotonin pathways as well as microbiota-derived indole pathways, which together regulate mucosal barrier integrity and immune homeostasis (Zelante et al., 2013; Yano et al., 2015).

Several limitations of this study should be acknowledged. First, the gut microbiota and metabolomic analyses were conducted at a single time point and do not provide strain-level functional information or absolute metabolite quantification, precluding definitive conclusions regarding causal relationships within specific microbiota–metabolite pathways. Further studies are therefore required to validate these associations. Second, KYNA co-treatment represents a pharmacological intervention strategy. Although disease severity was lower in mice receiving KYNA co-treatment, other metabolic abnormalities and extraintestinal immune mechanisms may also contribute to the observed phenotype, and their specific roles warrant further investigation. Third, this study did not include a non-EAU group treated with high-dose CAP. Without a non-immunized CAP-treated group, it is difficult to determine which intestinal changes are direct pharmacological consequences of high-dose CAP and which reflect interactions specific to the EAU inflammatory state. Fourth, the source of IL-17 should be assessed in the spleen, including the proportion of Th17 cells, to further clarify the immune response induced by CAP.

## 5 Conclusion

High-dose CAP exposure during EAU induction was associated with greater ocular inflammation, concurrent intestinal dysregulation, and elevated IL-17. KYNA co-treatment partially attenuated ocular and intestinal abnormalities, suggesting a potential link between reduced KYNA availability and the CAP-associated disease phenotype in EAU. Further studies using targeted metabolomics, microbiota transplantation, and pathway-specific interventions are required to establish the causal relationships underlying these findings.

## Data Availability

The datasets presented in this study can be found in online repositories. The names of the repository/repositories and accession number(s) can be found in the article/supplementary material.

## Glossary

16S rRNA: 16S ribosomal RNA
AhR: Aryl hydrocarbon receptor
APP/PS1: Amyloid precursor protein/presenilin 1
ARRIVE: Animal Research: Reporting of *In Vivo* Experiments
ARVO: Association for Research in Vision and Ophthalmology
BSA: Bovine serum albumin
CAP: Capsaicin
CFA: Complete Freund’s adjuvant
DAPI: 4′,6-diamidino-2-phenylindole
DEM: Differential metabolite
DMSO: Dimethyl sulfoxide
DNA: Deoxyribonucleic acid
EAU: Experimental autoimmune uveitis
EDTA: Ethylenediaminetetraacetic acid
ELISA: Enzyme-linked immunosorbent assay
FDR: False discovery rate
GPR35: G protein-coupled receptor 35
GSEA: Gene set enrichment analysis
H&E: Hematoxylin and eosin
HRP: Horseradish peroxidase
IL-1β: Interleukin-1 beta
IL-17: Interleukin-17
IL-17A: Interleukin-17A
IL-22: Interleukin-22
IRBP₁–₂₀: Interphotoreceptor retinoid-binding protein peptide 1–20
KEGG: Kyoto Encyclopedia of Genes and Genomes
KYNA: Kynurenic acid
LC–MS: Liquid chromatography–mass spectrometry
LDA: Linear discriminant analysis
LEfSe: Linear discriminant analysis effect size
MUC2: Mucin 2
NLRP3: NOD-like receptor family pyrin domain-containing 3
PCA: Principal component analysis
PCoA: Principal coordinate analysis
PE150: Paired-end 150-bp sequencing
PBS: Phosphate-buffered saline
PTX: Pertussis toxin
QIIME 2: Quantitative Insights Into Microbial Ecology 2
RNA: Ribonucleic acid
RNA-seq: RNA sequencing
SEM: Standard error of the mean
SPF: Specific pathogen-free
Th1: T helper 1 cell
Th17: T helper 17 cell
TNF-α: Tumor necrosis factor alpha
Treg: Regulatory T cell
TSA: Tyramide signal amplification
UPLC: Ultra-performance liquid chromatography
VKH: Vogt–Koyanagi–Harada disease
ZO-1: Zonula occludens-1
